# Application of Physiologically Based Pharmacokinetic (PBPK) Modeling Within a Bayesian Framework to Identify Poor Metabolizers of Efavirenz (PM), Using a Test Dose of Efavirenz

**DOI:** 10.3389/fphar.2018.00247

**Published:** 2018-03-27

**Authors:** Manoranjenni Chetty, Theresa Cain, Janak Wedagedera, Amin Rostami-Hodjegan, Masoud Jamei

**Affiliations:** ^1^Simcyp Ltd. (Certara), Blades Enterprise Centre, Sheffield, United Kingdom; ^2^Manchester Pharmacy School, Manchester University, Manchester, United Kingdom

**Keywords:** poor metabolisers of efavirenz, test dose of efavirenz, serious adverse effects to efavirenz, PBPK modeling with a Bayesian framework, CYP2B6 PMs

## Abstract

Poor metabolisers of CYP2B6 (PM) require a lower dose of efavirenz because of serious adverse reactions resulting from the higher plasma concentrations associated with a standard dose. Treatment discontinuation is a common consequence in patients experiencing these adverse reactions. Such patients benefit from appropriate dose reduction, where efficacy can be achieved without the serious adverse reactions. PMs are usually identified by genotyping. However, in countries with limited resources genotyping is unaffordable. Alternative cost-effective methods of identifying a PM will be highly beneficial. This study was designed to determine whether a plasma concentration corresponding to a 600 mg test dose of efavirenz can be used to identify a PM. A physiologically based pharmacokinetic (PBPK) model was used to simulate the concentration-time profiles of a 600 mg dose of efavirenz in extensive metabolizers (EM), intermediate metabolizers (IM), and PM of CYP2B6. Simulated concentration-time data were used in a Bayesian framework to determine the probability of identifying a PM, based on plasma concentrations of efavirenz at a specific collection time. Results indicated that there was a high likelihood of differentiating a PM from other phenotypes by using a 24 h plasma concentration. The probability of correctly identifying a PM phenotype was 0.82 (true positive), while the probability of not identifying any other phenotype as a PM (false positive) was 0.87. A plasma concentration >1,000 ng/mL at 24 h post-dose is likely to be from a PM. Further verification of these findings using clinical studies is recommended.

## Introduction

Efavirenz (EFV) is a potent non-nucleoside reverse transcriptase inhibitor (NNRTI) used in first line therapy of HIV-1 infection (Young et al., 1995). As part of combined antiretroviral therapy (cART), a standard oral dose of 600 mg daily had been prescribed. Recent analysis of the ENCORE1 study, where 400 mg daily (QD) was compared with 600 mg QD in treatment of EFV naive patients (no genotyping), showed that a 400 mg dose was not virologically inferior to a 600 mg dose as assessed at 48 weeks of treatment (Dickinson et al., 2015). A standard dose of 400 mg QD rather than 600 mg QD has been recommended to minimize adverse reactions and reduce treatment costs.

While being highly effective against the HIV-1 virus, EFV may be associated with neuropsychiatric effects in more than 50% of patients who receive a 600 mg dose, due to high plasma concentration (Marzolini et al., 2001; Pérez-Molina, 2002; Hawkins et al., 2005). These side effects may range from light-headedness, headache, dizziness, anxiety, insomnia, agitation, and impaired concentration to confusion, amnesia, frequent nightmares, depression, suicidal tendencies, and hallucinations (Marzolini et al., 2001; Núñez et al., 2001; Gallego et al., 2004; Haas et al., 2004; Hawkins et al., 2005). Symptoms usually appear soon after treatment initiation and disappear progressively a few weeks after (Marzolini et al., 2001; Dickinson et al., 2015). However, such side-effects may lead to discontinuation of therapy. An appropriate dose reduction in such patients is likely to prevent the CNS side effects that lead to poor compliance and treatment failure.

Large interpatient variability in plasma concentrations corresponding to a standard dose of EFV have been reported (Marzolini et al., 2001; Csajka et al., 2003; Kwara et al., 2009a; Siccardi et al., 2012). A key determinant in the dose to plasma concentration relationship was shown to be CYP2B6 metaboliser phenotype (Ward et al., 2003; Haas et al., 2004; Zanger et al., 2007; Kwara et al., 2009a; Sánchez et al., 2011; Maimbo et al., 2012; Meng et al., 2015). EFV is metabolized predominantly by the polymorphic CYP2B6, with a minor contribution from CYP3A4, CYP2A6, CYP1A2, and UGT2B7 (Ward et al., 2003; Desta et al., 2007; Rotger et al., 2007; Bélanger et al., 2009; Ogburn et al., 2010). Clinical studies have shown that plasma concentrations of EFV are significantly higher in EFV poor metabolisers (PM—CYP2B6^*^6/^*^6 genotype), compared with extensive metabolisers (EM—CYP2B6^*^1/^*^1 genotype), or intermediate metabolisers (IM—CYP2B6^*^1/^*^6 genotype), increasing the likelihood of CNS side effects associated with a standard 600 mg or 400 mg dose of EFV (Marzolini et al., 2001; Haas et al., 2004; Mukonzo et al., 2009; Maimbo et al., 2012). Lower doses (200 mg) of EFV are reported to be more appropriate in individuals who are PMs (Gatanaga et al., 2007; Sánchez et al., 2011; Siccardi et al., 2012). Early identification of CYP2B6 PMs will therefore assist prescribers in dose selection for such patients. While genotyping is a useful tool to achieve this, it is not affordable in under-resourced communities such as in many parts of Africa, where the prevalence of HIV is high. The cost of importing genotyping kits are unaffordable in such communities. However, EFV plasma concentration measurements are performed by some local laboratories. The question about the potential utility of a plasma concentration of EFV to differentiate between EFV metaboliser phenotypes was then raised, since a good correlation between phenotype, and plasma concentrations of EFV have been reported. If a single plasma concentration can be used to predict a PM in under-resourced communities, it would be a cost-effective means for prescribers to identify patients who may require a lower dose of EFV and thereby avoid treatment discontinuation by such patients.

The aim of this study was to determine whether a single plasma concentration can be useful in identifying the PM metaboliser phenotypes after a 600 mg test dose of EFV, using PBPK modeling with a Bayesian framework.

## Methods

### Overview of modeling and simulations

The Simcyp (V13 R2) population-based simulator (Simcyp, Sheffield, UK) was used for simulation of the PK of EFV in EM, IM, and PM subjects in a virtual healthy Caucasian population. Based on the clinical data (Siccardi et al., 2012; Xu et al., 2013) that was used for comparison, EM, IM, and PM represented the 516 GG genotype, 516 GT genotypes, and the 516 TT genotype, respectively. Details of the Simcyp population-based simulator, including data inputs required, algorithms used for calculation of PK parameters, methods of generation of population variability and applications supporting the performance verification of the simulator have been described previously (Rostami-Hodjegan and Tucker, 2007; Jamei et al., 2013). The simulations of concentration-time profiles as well as the predicted PK parameters of EFV in EM, IM, and PM subjects were compared with clinically observed data to verify the suitability of the models.

Simulated plasma concentrations were used in the Bayesian framework to identify the optimal sampling time and determine the probability of identifying a PM. Clinically observed data were used for further model verification.

### EFV PBPK model

The PBPK model for EFV was based on a published model (Xu et al., 2013), that represented a refinement of previously developed PBPK models (Rekić et al., 2011; Siccardi et al., 2012). A whole body PBPK distribution model with a compartmental absorption transit model was used to predict the PK of EFV in EM, IM, and PM phenotype subjects. CYP2B6 abundance data corresponding to the EM, IM, and PM phenotypes were obtained from Lang and coworkers (Lang et al., 2001).

#### EFV simulations for model verification

Simulations were run using 10 trials of 30 virtual healthy Caucasian subjects (50% female) in the EM, IM, and PM groups, for verification of the models. For the single dose simulations a 600 mg dose of EFV was used and the duration of the study was 72 h. Plasma concentration profiles and PK parameters (AUC_(0−∞)_, CL, Cmax) were compared with reported clinical data (Siccardi et al., 2012; Xu et al., 2013). Plasma concentration-time points were extracted from the figures in these publications by digitization, using the graph digitizer GetData (version 2.2).

Comparison of the simulated concentration-time profiles with the clinical data were checked visually. For acceptable recovery of the clinical data by the model, observed data points were expected to fall within the 5 and 95% percentiles of the simulations.

Predicted and observed PK parameters were also compared. An acceptable prediction error (predicted mean value:observed mean value) of 2-fold (Abduljalil et al., 2014) was used when comparing PK parameters.

#### EFV simulations for development of the bayesian model

After verification of the PBPK model in recovering clinical data corresponding to a single 600 mg dose of EFV, concentration-time profiles for 5,000 (10 trials of 500 subjects with 50% females) healthy Caucasian virtual subjects were simulated with each of the EM, IM, and PM phenotypes. Concentration-phenotype pairs corresponding to 2, 4, 8, 12, and 24 h post dose were used for development of the Bayesian model to predict the optimal sampling time to differentiate between EM, IM, and PM phenotypes. Selecting a sampling time that corresponded to the highest probability of identifying a PM was the primary objective of this Bayesian model development.

#### EFV simulations for verification of the bayesian model

After determining the optimal time for correctly predicting a PM phenotype, the reproducibility of the optimal Bayesian model was verified using simulated (from PBPK model) concentration-time profiles for a further 100 subjects approximately reflecting the phenotype frequency in Caucasians (46% EM; 38% IM; 26% PM) (Arab-Alameddine et al., 2009) after a single 600 mg dose of EFV. The scientist testing the Bayesian model was blinded to the phenotype associated with the plasma concentrations for the optimal time sample.

#### EFV clinical data used for final verification of the bayesian model

Clinical data were used to further verify the Bayesian model. 24 h plasma concentration- phenotype pairs from 36 subjects (that were not used for PBPK model verification) were used for the final verification of the Bayesian model. These clinical data were kindly shared by Dr. Marco Siccardi from the University of Liverpool, United Kingdom. These data formed part of the study that was published previously by this research group (Siccardi et al., 2012). Apart from dosing details and confirmation of the absence of any interacting drugs, no additional details of the subjects were supplied.

### Development and verification of the bayesian model

A Bayesian framework was used to calculate the probability of predicting the EFV metaboliser phenotype given a single plasma sample for an individual, following a 600 mg dose of EFV. This was derived for samples at 2, 4, 8, 12, and 24 h after dosing using data for the 5000 simulated individuals

At each time point the probability of each concentration value given the phenotype was calculated by counting the number of a particular rounded concentration (nearest 100) value at each time point for a particular phenotype and dividing by the total number of simulated concentrations at the same time point for the same phenotype.

If *C*_*it*_ is defined as the concentration value *i* (*i* = 1,….*n*) measured at time *t*, and *e*_*j*_ is defined as the enzyme with phenotype *j* where *j* = *PM, IM, EM*, the probability of observing concentration *C*_*it*_ at time *t* given phenotype *e*_*j*_ is calculated using Equation (1) below:

(1) $$\begin{array}{r} P\left(C_{it}|e_{j}\right)=\frac{Number\;of\;individuals\;with\;phenotype\;e_{j}\;and\;observed\;concentration\;C_{it}\;at\;time\;t}{Total\;number\;of\;observed\;concentrations\;for\;phenotype\;e_{j}\;at\;time\;t} \end{array}$$

Using the previously calculated probability from Equation (1) and Bayes theorem, the probability *P*(*e*_*j*_|*C*) of the phenotype *e*_*j*_ given the concentration *C*_*it*_ observed at time *t* can be calculated using Equation (2):

(2) $$\begin{array}{r} P\left(e_{j}|C_{it}\right)=\frac{P\left(e_{j}\right)P\left(C_{it}|e_{j}\right)}{P\left(C_{it}\right)} \end{array}$$

*P*(*e*_*j*_) is the prior probability of phenotype *e*_*j*_ where *j* = *PM, IM, EM*. As 5,000 individuals were simulated for each phenotype, the three phenotypes are all equally likely. Therefore, $P\left(e_{j}\right)=\frac{1}{3}$ for all phenotypes. *P*(*C*_*it*_|*e*_*j*_) is the probability of a concentration at a given sampling time given the phenotype and $P\left(C_{it}\right)=\underset{j}{\sum }P\left(CC_{it}|e\right)P\left(e_{j}\right)$ is the probability of concentration *C*_*it*_ at time *t*, divided by phenotypes.

These calculations result in a probability of each phenotype given the observed concentration.

Bayes decision theory was then used to determine the most likely phenotype for a particular concentration at each time point. The predicted phenotype for a given concentration was determined by maximizing the probability *P*(*e*_*j*_|*C*_*it*_) over all phenotypes *e*_*j*_ where *j* = *PM, IM, EM*. For a given concentration, *C*_*it*_, the predicted phenotype was determined using Bayes decision theory, where the most likely phenotype was determined to be *e*_*i*_ if

(3) $$\begin{array}{r} P\left(e_{i}|C_{it}\right)>P\left(e_{j}|C_{it}\right)\;for\;all\;j\neq i \end{array}$$

The reliability of the predictions was assessed by calculating the probability of correctly predicting each phenotype (true positive) and the probability of correctly rejecting each phenotype (true negative). The true positive, *P*(+|+), and true negative, *P*(−|−), values were calculated at each time point and for each phenotype using:

(4) $$\begin{array}{rcl} P\left(+|+\right) & = & \frac{Number\;correctly\;predicted\;phenotype\;e_{i}\;}{Number\;of\;indivuals\;with\;phenotypes\;e_{i}} \end{array}$$

(5) $$\begin{array}{rcl} P\left(-|-\right) & = & \frac{Number\;correctly\;predicted\;not\;to\;be\;phenotype\;e_{i}\;}{Number\;of\;indivuals\;not\;phenotype\;e_{i}} \end{array}$$

The true positive and true negative probabilities are important in assessing how good the model is at predicting a phenotype or the sensitivity and specificity of the model. The value *P*(+|+) should be close to 1 to ensure that phenotypes are correctly predicted, thus indicating good sensitivity of the model. However the value of *P*(−|−) should also be high to minimize the chance of incorrectly determining the wrong phenotype, thus indicating that the model shows good specificity. For example, if *P*(+|+) is high and *P*(−|−)is low for a particular phenotype it could suggest that a large proportion of the individuals are determined to be the same phenotype and other phenotypes are never predicted, resulting in a model that is not good at distinguishing between the phenotypes.

To determine the optimal sampling time point for correctly predicting each phenotype the true positive probability *P*(+|+) and false positive probability *P*(+|−) = 1−*P*(−|−) was calculated for each time point and phenotype. The values for each time point were then plotted for each phenotype as a ROC curve to determine the optimal sampling time for correctly predicting each phenotype. The optimal time point is the point on the graph which has a combination of both a value of *P*(+|+) as close to 1 and *P*(+|−) close to 0. Different combinations of *P*(+|+) and *P*(+|−) values can determine points that are equally as effective at correctly predicting a phenotype. Once the optimal time point has been determined a graph of the probabilities defined in (2) for the probability of each phenotype given an observed concentration at the time point was plotted. Using this plot and Bayesian decision theory defined in equation (3) the range of likely concentrations were determined for each phenotype.

The predictive capacity of the final Bayesian model was tested using the clinical data taken at 24 h. A phenotype was predicted for the observed concentrations at each time point using the results from the Bayes decision theory analysis in equation (3).

These predictions were then compared with the actual observed phenotypes (that had been previously determined by genotyping) to assess the reliability of the model to predict phenotypes given a single concentration. The reliability of the predictions was assessed by calculating the probability of correctly predicting each phenotype (true positive) and the probability of correctly rejecting each phenotype (true negative).

## Results

### EFV PBPK models for EM, IM, and PM

**Figure 1:**
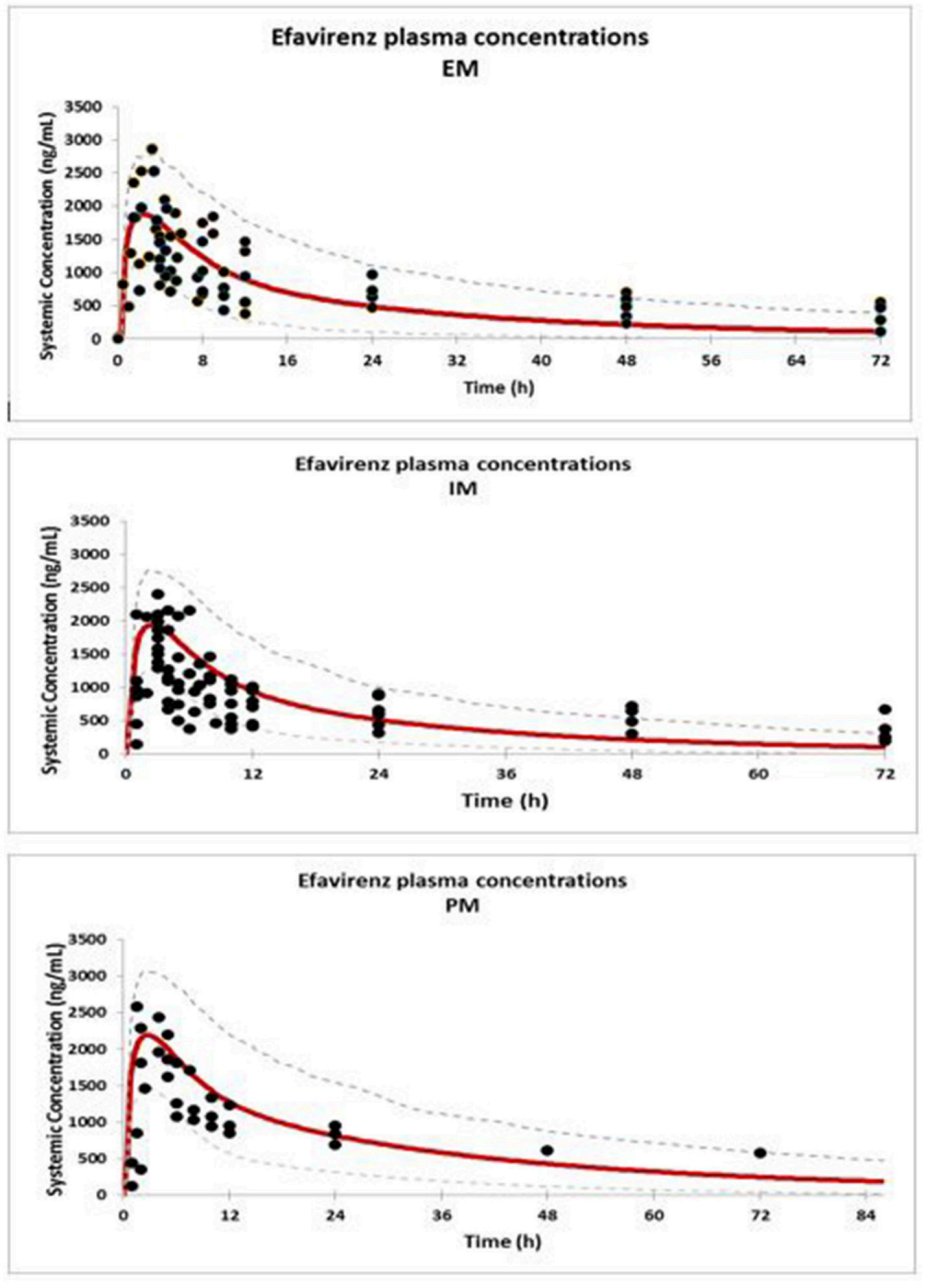
Comparison of predicted (solid red line = mean; dashed line = CI) and observed concentrations (Xu et al., 2013) (solid dots) of EFV in EM, IM, and PM.

Concentration-time profiles simulated for EFV in IM, EM, and PM are presented together with observed concentrations in Figure 1. Visual checks show good recovery of the clinical data by the PBPK models for each EFV metaboliser phenotype.

The predicted PK parameters for the three EFV metaboliser phenotypes together with the clinically observed data are presented in Table 1. The ratios of predicted mean values: observed mean values indicate an acceptable prediction of the PK parameters in all groups.

**Table 1:** Summary of the Predicted (Pred) and Observed (Obs) PK parameters in CYP2B6 EM, IM and PM.

**Parameter**	**Predicted (Mean and CI)**	Observed^*^ (Mean and CI)	Ratio Pred/Obs^*^	Observed^**^ (Mean ± *SD*)	Ratio Pred/Obs^**^
**EM**					
AUC_(0−∞)_ ng/L.h	66.8 (51.4–81.9)	68 (47–102)	0.98	79.8 ± 28.4	0.84
Cmax ng/mL	1850 (1755–1871)	1642 (1469-1916)	0.81	2300 ± 700	1.13
CL L/h	12.8 (9.7–13.7)	7.57 (4.89–12.53)	1.51	8.5 ± 3.4	1.69
**IM**					
AUC_(0−∞)_ ng/L.h	108.4 (105.2–109.7)	77 (63–99)	1.41	81.6 ± 33.7	1.33
Cmax ng/mL	1952 (1945–2077)	1878 (1376–2404)	1.06	1700 ± 500	1.15
CL L/h	6.9 (5.7–7.6)	7.14 (5.47–8.38)	0.97	8.3 ± 2.8	0.83
**PM**					
AUC_(0−∞)_ ng/L.h	153.2 (131.8–180.2)	123 (102–128)	1.25	101.7 ± 7.9	1.51
Cmax ng/mL	2135 (2048–2161)	2344 (1780–2522)	0.91	2400 ± 200	0.89
CL L/h	4.7 (3.9–6.6)	4.09 (3.90–4.55)	1.15	5.9 ± 0.5	0.79

* *Siccardi et al., 2012*.

** *Xu et al., 2013*.

These results indicate that the PBPK models are suitable for the prediction of the PK profiles of EFV in EM, IM, and PM.

### Bayesian model

**Figure 2:**
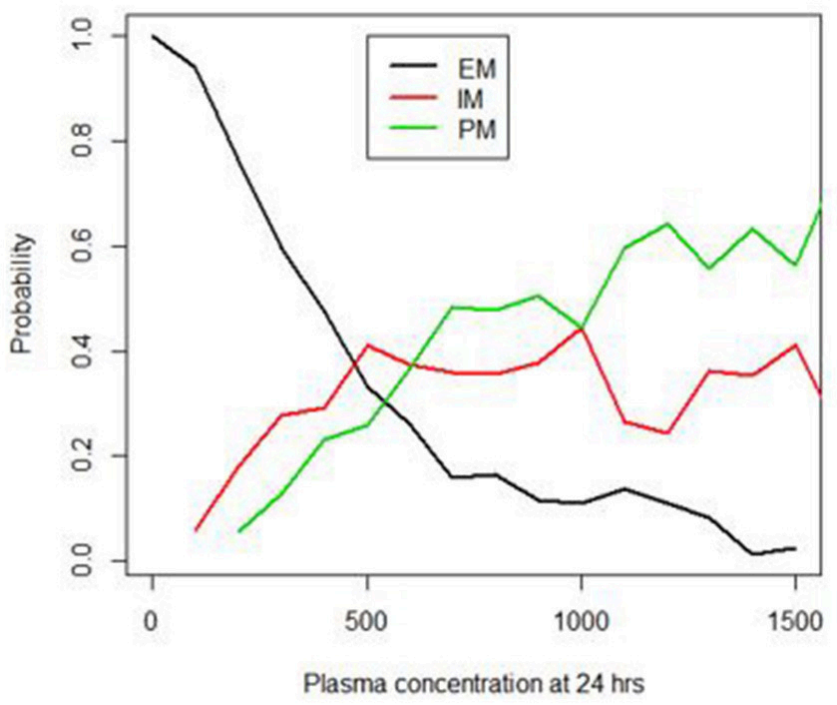
Probability of identifying the EM, IM and PM phenotypes using the 24 h plasma concentrations (ng/mL).

The developed Bayesian models showed that the 24-h sample following a single dose of EFV showed the highest probability (0.82) of differentiating the PM from the other phenotypes, when compared to the 2, 4, 8, and 12-h samples. Figure 2 shows a graph of the posterior probability of each phenotype given concentrations at 24 h after a single dose of EFV. This graph suggests that there is a high probability of predicting EM for concentrations less than 500 ng/mL. The probability of a PM increases above 0.6 when plasma concentrations exceed 1,000 ng/mL, while that for IM decreases below 0.3 and EM decreases below 0.2. This indicates that the probability of identifying a PM is highest with concentrations that exceed 1,000 ng/mL.

### Application of the model to predict the EFV EM, IM, and PM phenotypes using a 24 h plasma sample

To test the accuracy and reproducibility of the Bayesian model in correctly predicting a phenotype it was applied to a new set of observed/clinical data. Using Bayes decision theory, as described in the methods, and the probabilities presented in Figure 2 for each phenotype given a plasma concentration at 24 h, a phenotype was predicted for the observed concentration values of 36 new individuals. The predicted phenotypes were then compared with the actual phenotypes for each individual, that were determined previously by genotyping. Table 2 shows the probabilities of predicting each phenotype of the three phenotypes PM, IM, and EM given the true phenotype. As shown in the 3rd row of Table 2, the probability of correctly predicting a PM is high at 0.82 (4th column).

**Table 2:** Probability of predicting the true phenotype (EM, IM, or PM) from a 24-h plasma concentration.

**True phenotype**	**Probability of predicted phenotype**		
	**EM**	**IM**	**PM**
EM	0.57	0.36	0.07
IM	0.33	0.33	0.33
PM	0	0.18	0.82

Probabilities of correctly predicting a phenotype (true positive) and of correctly rejecting a phenotype (true negative) are presented in Table 3. In a perfect model these two probabilities would be both equal to 1 for all three phenotypes. For a PM, the sensitivity of the model or the probability of a correct prediction is 0.82, while the specificity or the probability of correctly predicting that the plasma concentration at 24 h is not from a PM is 0.87, suggesting that there is a very high probability of identifying PMs.

**Table 3:** Probability of predicting a true positive or true negative phenotype.

**Phenotype**	**Probability of prediction of a true positive**	**Probability of prediction of a true negative**
EM	0.57	0.85
IM	0.33	0.64
PM	0.82	0.87

## Discussion

It is evident from these results that the probability of correctly identifying a PM is >80% when the developed model is applied to a 24-h plasma sample following a 600 mg test dose of EFV. A plasma concentration >1000 ng/mL at 24 h post-dosing is most likely to be that of a PM. The application of such a model in a clinical setting to identify PMs can be very useful to clinicians who initiate antiretroviral therapy in patients diagnosed with the HIV infection. However, verification of these findings with more clinical data is recommended.

These results indicate that the sensitivity of the model to identify PMs is 82%, while the specificity is 87%. Using genotyping to identify patients who were likely to show very high plasma concentrations of EFV, the reported sensitivity was 46% while specificity was 97% (Swart et al., 2013). The higher sensitivity in this study suggests that this method could have some advantages over genotyping. A possible explanation for the difference is that genotyping does not consider additional variables such as concurrent medication, diet, and demographic characteristics which may impact on plasma concentrations.

Pharmacogenetics-informed dosing for EFV has been recommended to improve response and compliance and minimize toxicity and treatment failure, particularly in patients with a PM phenotype, where the risk of adverse drug effects is the highest (Marzolini et al., 2001; Haas et al., 2004; Gatanaga et al., 2007; Rotger et al., 2007; Sánchez et al., 2011; Siccardi et al., 2012; Meng et al., 2015). An EFV dose of 200 mg is reported to be more appropriate for a PM patient (Marzolini et al., 2001; Gatanaga et al., 2007; Sánchez et al., 2011; Siccardi et al., 2012), since 600 mg results in elevated plasma concentrations and the associated toxicity. In a clinical study with 12 Japanese PM subjects, Gatanaga and coworkers (Gatanaga et al., 2007) reduced the EFV dose from the standard 600 mg once daily (QD) to 400 mg QD. The viral load remained suppressed while the CNS symptoms improved. In 7 of these subjects, the dose was reduced further to 200 mg QD. The CNS symptoms disappeared and the viral load remained low, suggesting that the dose of 200 mg QD was more appropriate in these PM subjects. A population pharmacokinetic/pharmacogenetics study recommended doses of 600 mg QD, 400 mg QD, and 200 mg QD in EM, IM, and PM respectively (Sánchez et al., 2011). Similar EFV plasma concentrations were observed in the three groups based on the recommended doses. The impact of dose reduction in IM and PM on the pharmacokinetics and pharmacodynamics of EFV was analyzed using an *in vitro –in vivo* extrapolation (IV-IVE) model (Siccardi et al., 2012). Similar plasma concentrations and viral load suppression were predicted in the EM, IM and PM groups on 600 mg QD, 400 mg QD, and 200 mg QD, respectively. Thus, for optimum therapeutic outcomes, adequate evidence exists for treating PM with 200 mg QD rather than higher doses. The model developed in this study is expected to be useful in identifying PM patients where genotyping is not feasible.

Although a large number of virtual subjects were used for model verification, the number of clinical plasma concentrations (*n* = 36) used for verification was comparatively low. Further verification of the model with more clinical data and different populations will further enhance the model since EFV PK may differ in different populations. It is noteworthy that the Caucasian population model was used in this study but the frequency of the poor metaboliser polymorphisms varies between different population groups. The CYP2B6^*^6/^*^6 haplotype (516G>T and 785A>G) was reported to be the most frequent and functionally relevant variant across PM populations (Zanger et al., 2007). The CYP2B6^*^6/6 genotype is associated with reduced efavirenz clearance (Desta et al., 2007; Kwara et al., 2009b; Mukonzo et al., 2009; Maimbo et al., 2012), increased adverse effects (Haas et al., 2004; Gatanaga et al., 2007; Rotger et al., 2007; Wyen et al., 2011; Meng et al., 2015), and treatment discontinuation (Haas et al., 2004; Wyen et al., 2011). The frequency of this variant is 26% in Caucasians (Arab-Alameddine et al., 2009), 23% in Asian Americans, 62% in Papua New Guineans and 42% in West Africans (Mehlotra et al., 2006). The probability of correctly identifying a PM from a plasma concentration may be higher when the model is relevant to a population with a higher frequency of PM. Further model refinement using the different virtual populations can be considered in the future. PBPK models for HIV patients would be more appropriate, although it is currently unclear whether significant PK differences can be expected. Despite the use of healthy virtual patients, the PBPK models recovered the clinical data observed in HIV patients adequately.

The ability to identify a CYP2B6 PM using a single test dose of EFV may also have applicability to new drugs during development as well as drugs currently in use such as bupropion, where CYP2B6 contributes significantly to the metabolism and PK variability.

## Author contributions

MC: conceptualized and designed the study and contributed to the modeling; TC: contributed to the modeling; all the authors contributed to the writing of the manuscript.

### Conflict of interest statement

The authors declare that the research was conducted in the absence of any commercial or financial relationships that could be construed as a potential conflict of interest. All authors are employees of Simcyp Ltd. Simcyp has funded the use of the simulator. However, these funders had no role in study design, data collection and analysis, decision to publish, or preparation of the manuscript.
